# Identifying personal physiological data risks to the Internet of Everything: the case of facial data breach risks

**DOI:** 10.1057/s41599-023-01673-3

**Published:** 2023-05-08

**Authors:** Meng Wang, Yalin Qin, Jiaojiao Liu, Weidong Li

**Affiliations:** School of Journalism and Information Communication, Huazhong University of Science and Technology, Wuhan, Hubei Province China

**Keywords:** Science, technology and society, Cultural and media studies

## Abstract

Personal physiological data is the digital representation of physical features that identify individuals in the Internet of Everything environment. Such data includes characteristics of uniqueness, identification, replicability, irreversibility of damage, and relevance of information, and this data can be collected, shared, and used in a wide range of applications. As facial recognition technology has become prevalent and smarter over time, facial data associated with critical personal information poses a potential security and privacy risk of being leaked in the Internet of Everything application platform. However, current research has not identified a systematic and effective method for identifying these risks. Thus, in this study, we adopted the fault tree analysis method to identify risks. Based on the risks identified, we then listed intermediate events and basic events according to the causal logic, and drew a complete fault tree diagram of facial data breaches. The study determined that personal factors, data management and supervision absence are the three intermediate events. Furthermore, the lack of laws and regulations and the immaturity of facial recognition technology are the two major basic events leading to facial data breaches. We anticipate that this study will explain the manageability and traceability of personal physiological data during its lifecycle. In addition, this study contributes to an understanding of what risks physiological data faces in order to inform individuals of how to manage their data carefully and to guide management parties on how to formulate robust policies and regulations that can ensure data security.

## Introduction

We are now on the threshold of a new era of networking in which the Internet of Everything (IoE) can embrace IoE technologies, such as social networking, biometrics, multimedia and data mining, that can build relationships in various ways with terminals, platforms and users by connecting things, people, data and business processes (Adel and Michael, 2014). Given the relentless growth in IoE devices and their interaction with anybody with Internet access, virtually everything from physiological data to behaviour data is collected (Komendantova et al., 2021). The in-depth development of this data offers benefits to society for a variety of purposes in relation to authentication, border security, marketing, photo editing and social networking (Buckley and Hunter, 2011), but it also causes frequent data leakage events due to increased potential for a surveillance society (Buckley and Hunter, 2011).

In the past 5 years, a large number of serious personal data leakage incidents have occurred around the world. For example, in 2019 data leakage from Facebook in the United States impacted 540 million people. In addition, the SenseNets Horizon company leaked billions of facial data. These noncompliant and illegal data processing actions violate data protection laws (Raposo, 2022). Furthermore, in the IoE environment, personal physiological data has the characteristics of uniqueness, forever identification, replicability, irreversibility of damage and relevance of information. Leaked data violates individuals’ fundamental rights, such as the right to consent and deletion, privacy, equality and property (Brous et al., 2020; Raposo, 2022; Kindt, 2013). Data leaks can also lead to enormous, permanent damage to governments and enterprises. For example, in 2020 the Clearview AI data breach exposed the firm’s client list, resulting in bankruptcy (Hill, 2020).

To prevent data breaches and protect overall privacy, various countries have launched personal data protection mechanisms and promulgated laws and regulations on data security. A total of 142 countries issued data privacy legislation by 2020, of which the General Data Protection Regulation (GDPR) issued by the European Union (EU) has the greatest influence (Greenleaf and Cottier, 2020). Within the EU, the GDPR provides comprehensive and strict protection for facial data, and it gives individuals the right to informed consent and the right to delete. At the same time, countries and companies have also established the ethics compliance review to address accountability and transparency concerns and to mitigate risks. For example, IBM established an Ethics AI Board led by a Chief Privacy Officer for reviewing the ethics of technology rollouts (Almeida et al., 2022).

Although there are numerous efforts to address these concerns, data security and privacy risks have not been eradicated. The IoE era is aimed at connecting everything, and physiological data is easily accessible due to a large number of digital collecting devices around us with high-risk factors for data breaches. Moreover, the complexity of the cloud environment, the abundance of personal data and the lack of a unified framework of risk identification create real challenges to establishing a robust IoE scenario that acknowledges all these elements and comprises comprehensive data regulations (Al-Sharhan et al., 2019; Millard, 2013). Therefore, the identification of personal physiological data risks is the main task of current work and is also an important prerequisite to solve the security and privacy problems of personal physiological data.

Risk identification is the first step of risk management (Ozgur and Alkan, 2020; Mao et al., 2020). Only by choosing an effective risk identification method and correctly identifying personal physiological data risks can we actively take appropriate actions to avoid risks and ensure the safety of personal physiological data. The fault tree analysis method can identify the risks in the fault system and rank the importance of these risks (Ruijters and Stoelinga, 2015). Thus, by collecting, summarising and studying cases of facial data breaches, we can summarise the causes of personal physiological data breaches through the fault tree analysis method and provide insight into personal physiological data security.

The IoE context connecting everything leads to more risk sources for data breaches. Currently, a better and more profound analysis of the data breach risks is necessary due to the lack of an adequate understanding of privacy and risk awareness. Based on these myriad considerations, this study uses facial data breach accidents as the research objects and adopts fault tree analysis to systematically and comprehensively investigate the risks of physiological data breaches in line with the data lifecycle. The paper is structured as follows. The next section provides a literature review of relevant research. In the Methods section, we explain the concepts of fault tree analysis and data lifecycle and further outline the research steps. In the Data and Process section, we describe the data selection criteria and present a complete fault tree diagram of a facial data breach. In the Data Analysis and Results section, we analyse reports of the minimal cut set and reflect on the structural importance of basic events. This work’s contributions are presented in the Discussion section, after which the Conclusion section briefly summarises the main findings and implications.

## The generation and application of personal physiological data in the era of IoE

The IoE is a massive, complex network ecosystem consisting of various elements such as objects, digital devices, individuals, enterprises, governments and data resources through the support of digital platforms and digital processes (Li, 2020). Compared with the Internet of Things, the IoE has a wider range of connected objects and can interact more profoundly with people and the social environment (Adel and Michael, 2014), while the Internet of Things only connects things (sensors and devices) (Diega and Walden, 2016). In other words, the IoE is based on the Internet of Things but enables ‘things’ to be connected to any device and anyone using any path (network) and any service at any time (context) and anywhere (Spyros, 2018). In the IoE, the generation and application of personal physiological data mainly occur in the cloud, applications, and terminals.

In the traditional social environment, human’s natural attributes essentially belong to the category of privacy and are rarely collected digitally. Nevertheless, personal physiological data has been a crucial resource for data collection in the IoE environment. Physiological data is more reliable and measurable than abstract forms such as behavioural data because physiological data can reflect the human state intuitively and even can capture one’s state of mind. In a general sense, all human organs that constitute ‘physiological humans’ tend to be digitised.

Firstly, especially with the development of Internet of Nano Things (IoNT) technology, more human organs can be digitised and connected to the Internet. As the latest innovation of the IoE, IoNT is a network formed by integrating nanomachines (with a size range of 1–100 nm) with the existing traditional communication network and high-speed Internet (Anand et al., 2017). The IoNT can integrate nanosensors into various miniature objects to realise the intelligent perception of subtle environments, and each of its functional tasks is performed by ‘nano machines’. This allows the depth and breadth of the IoE to be greatly expanded and enables sensors to connect nano-scale devices as well. Thus, as long as the nanosensor is integrated into the networked object, it can be connected to and communicate with the Internet (Mainor and Patricia, 2019).

Secondly, the development of face recognition technology and human body recognition technology has allowed increasing amounts of facial data and human body data to be perceived and collected. In order to achieve ‘environmental intelligence’, the IoE needs to monitor and record the body movements, gestures, location, background and environment of human users (Miraz et al., 2015). Existing face recognition technology is mainly used to distinguish facial attributes. The facial data that can be recognised mainly includes the face frame, the key points of the face, the pose estimation of the face, the natural attributes of the face (i.e., whether one is wearing sunglasses, whether the face is covered, whether there is a beard), and emotional expressions (Kachur et al., 2020). Current human body recognition technology mainly carries out human key point detection, which can accurately estimate the 14 main key points of the human body in pictures or videos, such as elbows, wrists and shoulders (Xu et al., 2021). With multiple scenes, it can estimate multiple postures of standing, sitting and moving so as to detect and recognise postures (Patel et al., 2017).

Moreover, with the innovation of emotion recognition system and emotion computing technology, some new application platforms are trying to scientifically monitor individual emotions (surprised, happy, sad, angry, calm), psychological conditions and ideas by analysing the facial data or voice (Isiaka and Adamu, 2022; Kachur et al., 2020). For example, a mental health platform based on biofeedback and emotional computing attempts to capture and analyse the subtle colour changes of the facial capillaries through the mobile phone camera to achieve the monitoring of instantaneous heart rate and implicit respiration, and on this basis, it helps users resist psychological problems such as tension, anxiety, anger, depression, fatigue and insomnia (Li, 2020). In addition, digital platforms and technology companies integrate and correlate users’ physiological data through emotional calculation, in order to summarise the users’ emotional response to different stimuli, portray user images, and then put advertisements or carry out targeted promotions (Isiaka and Adamu, 2022).

Finally, with technological progress and the continuous generation of personal physiological data, the application of personal physiological data in medical treatment, finance, education and other fields has expanded rapidly. Taking the medical field as an example, a patient-centred health-data-sharing platform can harvest data from multiple personal digital devices to increase information accessibility and enhance the quality of health services (Dhruva et al., 2020; Al-Sharhan et al., 2019; Carvalho et al., 2019; Haraty et al., 2018; Gagnon et al., 2016). Medical and health wearable devices generally have built-in sensors for measuring human health that can automatically collect important data such as blood pressure, pulse, heart rate and body temperature. At present, smart bracelets, watches, glasses and athletic shoes can all perform this function. Overall, health data is generated from various sources, including electronic medical records, insurance claims, Internet of Things devices, and social media posts, which can be widely shared and applied, especially when it comes to disease treatment (Wang et al., 2017).

## Risks to personal physiological data in the IoE

The IoE has a wide range of connected objects and can interact in profound ways with people and the social environment (Mahoney and LeHong, 2012; Adel and Michael, 2014). The IoE has formed a huge user pool and has revolutionised social relations, information and knowledge sharing, as well as marketing opportunities (AlAlwan et al., 2017; Kapoor et al., 2018; Shiau et al., 2017). In this process, a variety of embedded devices or operating systems monitor an individual’s vital organs to continuously create physiological data second-by-second in order to provide various intelligent services, such as smartwatches used for evaluating sleeping quality (Sundar, 2020; Kwon et al., 2014). Hence, personal physiological data is becoming the most important data resource in the IoE era, and this data is widely digitised, recorded and tracked.

**Fig. 1:**
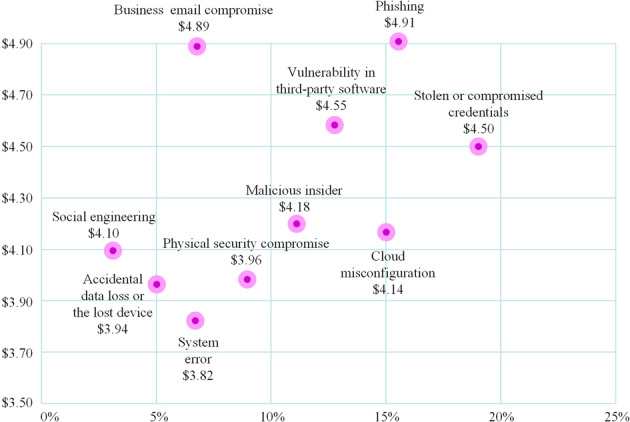
Cost of a Data Breach Report 2022 (from IBM Security, 2022). Note: Measured in USD millions. The most common initial attack vector in 2022 was stolen or compromised credentials, responsible for 19% of breaches in the study, at an average cost of USD 4.50 million.

However, efficiency and service improvements are often accompanied by increased security concerns (Sundar, 2020; Hadi et al., 2019; Hashem et al., 2016; Hossain and Dwivedi, 2014). Worse still, in the era of the IoE, technology companies, digital media platforms and the government—powered by intelligent collection equipment and information technology—are collecting physiological data on people’s digital daily lives in invisible ways. From the security inspection measures in public places such as airports and stadiums to ubiquitous cameras and face payment in supermarkets, physiological data is increasingly becoming an indispensable part of digital life that is almost beyond personal control (Lyon, 2017). A new survey has listed 10 breach risk sources and costs of a personal data breach (see Fig. 1), indicating that data breach is a complex and systematic problem. Particularly, physiological data is sensitive personal data and can face unprecedented threats once leaked (Jain et al., 2008). These threats abound in personal physiological data and may carry dire consequences for individuals, enterprises and nations (Brous et al., 2020). For individuals, physiological data breaches can cause privacy violations, property loss, genetic discrimination, security threats, and serious emotional and mental harm (Chin et al., 2012; Ji et al., 2018; Li et al., 2017; Kindt, 2013; Kilovaty, 2021). Breaches can also violate the fundamental rights of individuals which are enshrined in data protection laws, such as the right to informed consent, access and erasure, which can further damage traditional rights to one’s personal image, reputation and dignity. For enterprises, a variety of problems such as reduced credibility, damage to economic interests and lawsuits can result from a data breach (Kshetri, 2014). In the Clearview AI case, several major technology companies such as Google, Facebook, YouTube and Twitter publicly condemned Clearview AI following the data breach. On the national level, the impact of a physiological data breach can be far-reaching. For example, US credit enterprise Equifax was attacked by hackers who breached the data of 143 million US citizens, posing a serious threat to US national security. Undoubtedly, the serious threats of data breaches cause great costs to the damaged entities for mitigating its effect, such as repairing the vulnerability, training employees, legal costs, and more (Kilovaty, 2021), which has led to the urgency of the breach problem. Thus, a better and more profound analysis of the data breach risks is necessary due to the lack of an adequate understanding of threats and risk awareness.

## The application and uniqueness of facial data

The GDPR defines personal data as information related to an identified or identifiable natural person (data subject) and limits the processing of special types of personal data (personal sensitive information), including fingerprints, DNA, height, iris patterns, facial features and palm prints. As a special category of personal physiological data, facial data is a highly sensitive form of personal sensitive information. At the same time, facial data has assumed as an increasingly vital role in daily life due to the ubiquitous presence of face recognition technology, which is characterised by high acceptability, collectability and universality (Jain and Ross, 2004; Günther et al., 2017; Sepas-Moghaddam et al., 2019). Face recognition technology became especially successful as a result of the non-contact measures imposed during the COVID-19 pandemic. Scenarios in which face recognition technology has been applied include smart transportation (Karantzoulidis, 2019; Wollerton, 2019; Toor, 2017), device unlocking (Chmielewski, 2015; Finnegan and Kapo, 2018; Tengyuen, 2017), banking (Kan, 2015; Knight, 2017; Petroff, 2016), online retailer services (Association, 2013; Bates, 2017; Pearson, 2018; Romero, 2019), security checks (Wallace, 2018; West, 2019; Wolfe-Robinson, 2019; Oliver, 2019) and health care (Li et al., 2017; Ji et al., 2018).

Compared with other physiological data, facial data is unique. Firstly, facial data can be collected imperceptibly and used widely compared with fingerprint, palmprint or iris data due to non-contact and wide application of face recognition technology, thus facing more breach risks. Secondly, facial data has certain variability. The face can be changed through time, cosmetic surgery or light, resulting in more recognition difficulties and even recognition errors than that of fingerprint, palmprint or iris data. In addition, facial data is a special kind of personal sensitive data related with privacy, which not only covers the personality interests of the ‘face’, such as personal portrait, reputation and human dignity, but also reflects the additional economic value of data. Therefore, due to these similarities and differences, on the one hand, just like facial data breaches, other physiological data breaches can also be caused by similar risk sources and can produce similar serious consequences, such as privacy violations, property loss or physical danger (Sepas-Moghaddam et al., 2019; Ghaffary, 2019; Mehmood and Selwal, 2020; Nandakumar and Jain, 2015). On the other hand, compared with other physiological data, a facial data breach occurs more frequently and can lead to more harmful effects. Given that facial data is widely used and has high breach frequency, it is necessary to study facial data breach risks in order to inform individuals and data management parties of how to manage data carefully. Therefore, the identification of personal physiological data risks, especially facial data risks, is the main task of current work.

## Current research on risk identification

Risk identification is the foremost step of risk evaluation and management because timely and effective risk identification serves as the basis of overall safety protection. The results of risk identification can help to inform individuals, government agencies and enterprises of the appropriate measures they should take to address data security concerns. When identifying personal data breach risks, previous scholars have mainly adopted empirical and positive research approaches, and empirical research is more used comparatively. Existing empirical research has identified several risks leading to data breach accidents; these risks are primarily related to IoE devices, IoE technologies, third parties (enterprises, governments), user behaviour, malicious attackers and irregular operations (Palattella et al., 2016; Perlroth, 2016; Kshetri, 2014). Positive research methods are diverse and more often rely on questionnaires. Scholars have used questionnaires to collect information on hidden risks that endanger personal data security and have concluded that insufficient personal protection awareness and imperfect management systems are the reasons for personal information disclosure (Yan et al., 2018; Liu, 2015). As security incidents caused by physiological data breaches have raised concerns all over the world, individuals are increasingly aware of the importance of their physiological data and the possible risks of data breaches (Finnegan and Kapo, 2018). Scholars have also dedicated research efforts to the identification of personal physiological data breach risks (Ratha et al., 2001; Tuyls et al., 2007; Kumar et al., 2018). Previous studies have determined that the risks of physiological data breaches mainly come from indirect attacks and direct attacks (Jain et al., 2018; Smith et al., 2018; Wang et al., 2020). Indirect attacks are carried out from inside the data system, such as attacks by hackers, staff stealing data, template or database modifying, and other unauthorised accessing or activity (Tuyls et al., 2007). Direct attacks mainly refer to technology and device attacks, which are especially prevalent among immature face recognition technology and vulnerable collecting sensors.

In sum, existing research on the risks of personal data breaches has established a basic risk identification reference and listed several risk sources, but the research also has deficiencies. Firstly, particularly in the IoE era, primary data risks arise during data liquidity (Etzioni, 2015; Mayer-Schönberger and Cukier, 2013). To date, there has been little research on risks during data liquidity. Alshammari and Simpson (2017) suggested that understanding the data lifecycle as a representation of data liquidity could be the path to perceiving and tracing physiological data risks. Secondly, few studies have used systematic and objective methods to study and explain personal data breach risks and their logical relationship. A data breach is a kind of accident, but no one has tried to analyse data breaches with accident management methods. Thirdly, physiological data has multiple attributes, once leaked, the consequences are more serious than traditional personal data breaches. Moreover, facial data, as a special kind of personal physiological data, is widely used and has high breach risks. However, there is little research on the identification of personal physiological data breach risks, especially facial data breach risks. Finally, data breach is a widespread and complex problem involving multiple cases, and a single data breach case cannot fully explain comprehensive breach risks, and can no longer meet the research needs.

## Methods

There are many studies related to risk identification methods. The main methods of risk identification include fault tree analysis (Yuan et al., 2021), the Delphi method (Gephart et al., 2013), brainstorming (Feng et al., 2017), scenario analysis (Haab et al., 2010), checklist method (Ozgur and Alkan, 2020), Bayesian network analysis (Mao et al., 2020) and the analytic hierarchy process (Liang and Lin, 2017). Considering the complexity of data breaches as well as the effectiveness and reliability of conclusions, we selected fault tree analysis (FTA) as our method for the following reasons.

Firstly, the fault tree analysis method has a wide range of applications and a mature research system. It is a diagram and deductive procedure for safety and reliability analysis in which the causal chain leading to failure is explored using graphical tools (Ruijters and Stoelinga, 2015). It can determine the various combinations of system failures and human errors that can cause undesired events (referred to as top events) at the system level through logic gates (Kabir, 2017). In fault tree analysis, starting from the fault state of the system, we aim to comprehensively identify the risk causes leading to the top event, and we clarify the direct and potential events of the accident through in-depth analysis in a logical and hierarchical way. This method is particularly suited to the analysis of complex systems because it can effectively model a vast number of system components, and it is therefore widely used in the aerospace, energy, network information systems and construction fields.

Secondly, as a massive and complex system, the IoE contains a large amount of personal data. Within such a complex system, there are various factors that can lead to a data breach. Fault tree analysis can be used in complex systems to identify multiple risks caused by personal, administrative and environmental factors in an intuitive and logical way. It can specifically and clearly analyse the causes and propagation processes of faults, which is conducive to further risk management and data safety.

Thirdly, a data breach is a kind of accident, but no one has tried to analyse data breaches with accident management methods. The fault tree analysis method can explore the impact of each basic event on the accident through qualitative analysis so as to determine the priority of measures for the safety control of each basic event and to provide a timely and effective solution or reference for urgent data breach accidents. Therefore, the fault tree analysis method is feasible and appropriate for analysing personal data security problems.

Horton (1985) proposed the concept of an information lifecycle composed of a series of logical stages and maintained that this lifecycle is the natural law of information movement. In the context of information-centric domains, data is dynamic, changeable and ubiquitous, and it is subject to a variety of actions—including collection, storage, usage, transmission and destruction—by several actors for various purposes. These combined actions constitute a data lifecycle. The data lifecycle provides a means to classify, compare and construct data and provides a framework that can systematically and proactively identify and address risks during the various periods of a potential data breach. Likewise, any kind of physiological data undergoes the process of collection, storage, usage, transmission and destruction. In each period of the data lifecycle, a physiological data breach usually involves similar functions, purposes and participants. Therefore, physiological data breach risks can be generalised by taking a facial data breach event as the research object based on data lifecycle.

We regard facial data breaches as an undesired or top event, and we analyse facial data breaches through a case study based on a data lifecycle with five stages: data collection, data storage, data transmission, data usage and data destruction. We adopt fault tree analysis to determine the failure mode leading to facial data breach and the importance of various risk factors to the accident.

The procedure is performed stepwise, with the first step being a choice of the top event (T), in this case, facial data breach, which is an undesirable event associated with the facial data system. The next step is to determine all the secondary events classified into intermediate events (M) and basic events (X) by considering the data lifecycle and risk factors that can cause the top event (T). If two or more secondary events must both occur to cause the top event, these events will be linked in the tree by an ‘AND’ gate. According to this study, the top event T is caused by the three intermediate events of M_1_, M_2_ and M_3_ with the logic of the ‘AND’ gate. However, if either of the events will cause the top event, the secondary events will be linked to the top event by an ‘OR’ gate. Then, an ordering of causative events with a logical relationship will be generated until the fault tree diagram is presented. A further step involves sorting out the minimal cut sets of the fault tree and then calculating the structural importance. Finally, according to the results, we can identify the most significant vulnerabilities in the system and make effective recommendations for reducing the risks associated with those vulnerabilities. The terminologies and symbols in the fault tree diagram are explained in Table 1.

**Table 1:** Fault tree symbols (Barlow and Proschan, 1975).

Event symbol	Terminology	Description
	Top Event, T represents top event	The TOP event is the accident that is being analysed.
	Intermediate Event, Mi represents intermediate event	The INTERMEDIATE events are system states or occurrences that somehow contribute to the accident.
	Basic Event, Xi represents basic event	The BASIC event indicates a basic initiating event at the limit of resolution.
	Undeveloped Event	The UNDEVELOPED event is undeveloped because there we either lack information or the event is of no consequence.
Gate symbol	Terminology	Description
	AND Gate	The AND gate indicates that the output fault (drawn above the gate) only occurs if two (or more) input faults (drawn below the gate) occur.
	OR Gate	The OR gate indicates that the output fault occurs if at least one of the two (or more) input faults occur.

### Data and process

A personal data breach means a breach of security leading to the accidental or unlawful destruction, loss, alteration, or unauthorised disclosure of or access to personal data (Voigt and Von dem Bussche, 2017). Thus, a breach is more than just losing personal data. Facial data, as a sensitive data type that can be linked to eternal identity, can also have a detrimental impact on an individual or society if the data is breached. In order to identify and enumerate the risks leading to facial data breach and to list the casual events in the fault tree diagram, we collect cases from the China National Knowledge Internet (CNKI) newspaper database, China Search (chinaso.com), Baidu News and Google News using the keywords ‘facial data breach’, ‘facial data accident’, ‘facial data abuse’, ‘face recognition’ and ‘facial data’. We select 22 facial data breach cases that have occurred since 2019 as the research objects. The selected cases meet the following criteria.

1) *Typicality*: The case has attracted extensive attention and discussion across society and has a certain social influence and representativeness.

2) *Availability*: As a method suitable for complex systems, fault tree analysis requires multi-channel and multi-type data to explain and support a case. Therefore, we generally select current cases that have been publicly reported by the media or studied in academic research to obtain detailed supporting materials. These cases have a complete and clear development background, which can ensure the validity of the research.

3) *Heterogeneity*: Facial data breach accidents are pervasive issues, and therefore similar, homogeneous cases that make it difficult to extract variables. As such, cases with a range of fields, places of occurrence and subjects involved shall be considered.

The breach cases are shown in Table 2. The details of these cases can be found in Appendix 1.

**Table 2:** Facial data breach cases.

No.	Case	No.	Case
1	New data breach has exposed millions of fingerprint and facial recognition records	12	Students sue online exam proctoring service Proctor U for biometrics violations following data breach
2	Clearview AI data breach exposes facial recognition firm’s client list	13	Hackers breach thousands of security cameras, exposing Tesla, Jails, hospitals
3	In 2019, it was reported that hackers breached Apple’s iPhone FaceID user authentication in just 120s	14	The SenseNets Horizon company leaks billions of facial data.
4	A group of hackers breached popular surveillance and facial recognition camera company, Verkada	15	IBM collects online photos without consent, the individual find it impossible to delete their facial data
5	Facial data of thousands of Chinese students has been leaked	16	Everalbum collects facial pictures illegally
6	Residential neighbourhoods across China are adopting facial recognition	17	Facial data leakage of students in some schools in Sichuan and Gansu
7	LLC, transferred copies of CBP’s biometric data, such as traveller images, to its own company network	18	Zhang Fu and others violated citizens’ personal information and fraud
8	Biometric information exposed in slot machine operator data breach	19	Abuse of face recognition in stores such as Kohler bathroom and BMW
9	7-Eleven breached customer privacy by collecting facial imagery without consent	20	Twinning’s personal information breach
10	Consumer-facing companies still have few incentives to stop data breaches, and that’s a national security concern	21	The biometric data leakage occurred to Suprema
11	Massive biometric data breach found in system used by banks and Met police	22	Abuse of face recognition technology in housing sales industry (29 administrative punishment cases)

This paper analyses and extracts the risks of each facial data breach accident according to two aspects: (1) The causes or risks of facial data breaches are directly pointed out in the news report. For example, in the passenger information breach of U.S. Customs and Border Protection (CBP) in May 2019, the causes for the data breach were that a subcontractor violated the protocol policy and transmitted photos of passengers and license plates to a home network without authorisation; this database was then attacked by hackers. (2) The other aspect is based on the expert analysis of a news report. For example, February 2019 witnessed a facial data breach accident in SenseNets Horizon. The direct causes of the breach were ‘no password protection for the internal database’ and an ‘access restriction configuration fault’. However, some experts added that the company had insufficient security awareness and there are loopholes in its network security compliance.

This paper starts from the top event and then determines the intermediate events, basic events and the logical relationship between them level by level based on the risks of facial data breach cases identified via the aspects of direct cause and expert analysis. Three PhD students initially sort the risks based on the two aspects and then discuss them, and one professor finally checks and confirms the risks. Overall, the validity of the selected cases and collective intelligence contribute to the accuracy and completeness of the identified risks and the logical relationship. After that, to simplify the fault tree diagram, we code each event and present an event code list, as shown in Table 3.

**Table 3:** List of events at all levels.

Code	Event	Code	Event
*T*	Facial data breach	*X*_12_	Web crawler collection
*M*_1_	Risk caused by an individual	*X*_13_	Illegal collection by offline face recognition camera
*M*_2_	Risk during data management	*X*_14_	App illegally opens cameras of mobile phone and other devices to collect data
*M*_3_	Supervision absence	*X*_15_	The enterprise has ineffective security measures and insufficient data protection capacity
*M*_4_	Individual’s insufficient security awareness	*X*_16_	Insufficient safety education
*M*_5_	Individual’s unsafe actions	*X*_17_	The database without password
*M*_6_	Risk during data collection	*X*_18_	Simple username and password
*M*_7_	Risk during data storage	*X*_19_	Access rights configuration error
*M*_8_	Risk during data transmission	*X*_20_	Incorrectly configured databases
*M*_9_	Risk during data usage	*X*_21_	Not desensitised core data
*M*_10_	Risk during data destruction	*X*_22_	Low server security
*M*_11_	The supporting policies not keeping up with technology	*X*_23_	Equipment lost
*M*_12_	Data is collected randomly	*X*_24_	The physically compromised storage device
*M*_13_	Insufficient awareness of safety responsibilities	*X*_25_	The equipment is not updated in time
*M*_14_	Unsafe database	*X*_26_	Interface attack
*M*_15_	Illegal use	*X*_27_	Management account is hijacked
*M*_16_	Defective collection equipment	*X*_28_	Malware attack
*M*_17_	Low difficulty of collection	*X*_29_	Data outsourcing, hosted by a third-party company
*M*_18_	Illegal collection	*X*_30_	Data exiting
*M*_19_	Database security protocol flaw	*X*_31_	Data is captured during data transmission
*M*_20_	Vulnerable physical storage device	*X*_32_	Commercial companies actively sell data
*M*_21_	Database is attacked	*X*_33_	Commercial companies went bankrupt and data was resold
*M*_22_	Illegal actions by data management party	*X*_34_	Operators steal data
*M*_23_	Illegal actions by the third party	*X*_35_	Remote access by operators leaves data on other devices
*M*_24_	Hacker attack	*X*_36_	Illegal storage of data by third parties
*X*_1_	Personal greed for small gains	X_37_	Illegal transfer of copies by third parties
*X*_2_	Incautious about the related products	*X*_38_	The third-party did not delete the data as required
*X*_3_	Individuals lack deletion consciousness	*X*_39_	Illegal trade
*X*_4_	Individuals use simple password	*X*_40_	The data user has not fully considered or prepared for the risk
*X*_5_	Individuals upload facial data for entertainment actively and casually	*X*_41_	The management party failed to delete the data as required
*X*_6_	Individuals download unapproved Apps	*X*_42_	Incomplete data deletion
*X*_7_	Immature face recognition technology	*X*_43_	Lack of laws and regulations
*X*_8_	The device is invaded by Trojan Horse programme	*X*_44_	Lack of collection standards and specifications
*X*_9_	The collection device is invaded physically	*X*_45_	Lack of implementation rules for face recognition technology
*X*_10_	Rough collection method	*X*_46_	The review mechanism of science and technology ethics is incomplete
*X*_11_	Concealed collection means	*X*_47_	Lack of data deletion mechanism

**Fig. 2:**
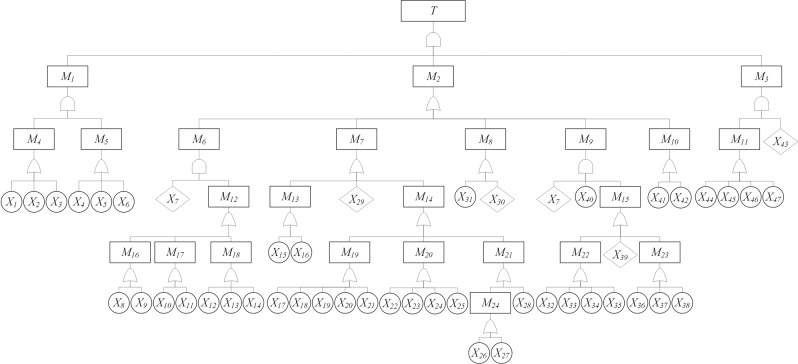
Fault tree diagram of a facial data breach. *T* is caused by *M*, *M* is caused by *X*.

Then, in line with the event code list (Table 3), the events of each level are connected by logic gates according to the logical relationship between the top event (*T*), intermediate event (*M*) and basic event (*X*) sorted from the 22 selected breach cases so as to present the fault tree diagram of facial data breach level by level (as shown in Fig. 2). The first level is the top event (*T*), the second level is the intermediate event (M), the third level is the basic event (*X*). Overall, *T* is caused by *M*, *M* is caused by *X*. *T*, *M* and *X* are all exacted from the 22 cases.

**Fig. 3:**
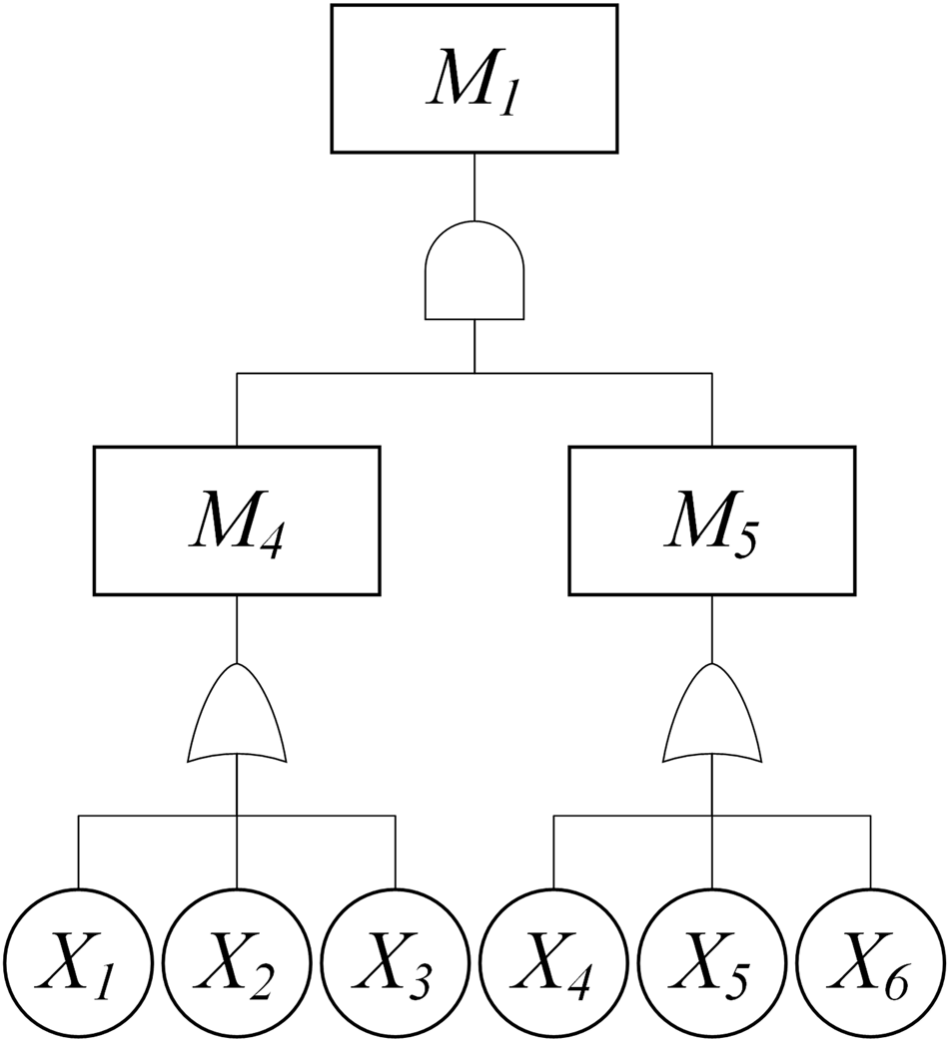
Part of fault tree diagram of facial data breach. *M*_1_ is caused by *M*_4_ and *M*_5_. *M*_4_ is caused by *X*_1_ or *X*_2_ or *X*_3_. *M*_5_ is caused by *X*_4_ or *X*_5_ or *X*_6_.

Facial data breach is always caused by various factors. These factors can form some causal chain, such as ‘Individual’s insufficient security awareness’ can be caused by ‘Personal greed for small gains’ or ‘Incautious about the related products’ or ‘Individuals lack deletion consciousness’. The following example will clearly present how this process could be applied when performing a fault tree analysis on the facial data breach (see Fig. 3):

The event to analyse: *M*_1_ (Risk arisen by individual)

The contributing factor 1: *M*_4_ (Individual’s insufficient security awareness)

Possible causes for *M*_4_: *X*_1_ (Personal greed for small gains), *X*_2_ (Incautious about the related products), *X*_3_ (Individuals lack deletion consciousness)

The contributing factor 2: *M*_5_ (Individual’s unsafe actions)

Possible contributing factors to *M*_5_: *X*_4_ (Individuals use simple password), *X*_5_ (Individuals upload facial data for entertainment actively and casually), *X*_6_ (Individuals download unapproved APPs)

In this example, factor 1 (*M*_4_) and factor 2 (*M*_5_) combined could ultimately cause *M*_1_, represented by an ‘AND’ gate, meaning the two events always occur together which could lead to *M*_1_. Each gate is then expanded level by level to ultimately identify the lowest-level causes.

### Data analysis and results

#### Analysis of minimal cut set

In the structural analysis of a fault tree diagram, the minimal cut set (MCS) is the minimal basic event combination leading to the occurrence of a top event, and this reflects the risk of the system. The number of MCSs in the fault tree is equal to the kinds of possibilities leading to the top event. In other words, the more the MCSs, the more the paths of accidents, and therefore the riskier the system. The Fussell-Vesely algorithm is usually adopted to calculate MCSs: starting from the top event, the upper-output event of the logic gate is explained by the lower-input event level by level according to the different logical relations of the intersection. If it is an ‘OR’ gate, the union of input events (represented by multiplication) is presented. If it is an ‘AND’ gate, the intersection of input events (represented by addition) is presented. This continues until all events are replaced with bottom events (basic events). In this way, the final algorithm result is composed of several MCSs. According to Fig. 1, the top event *T* is caused by the three intermediate events of *M*_1_, *M*_2_ and *M*_3_ with the logic of the ‘AND’ gate. In this way, we obtain the formula of the top event as *T* = *M*_1_ × *M*_2_ × *M*_3_. Subsequently, *M*_1_ is caused by *M*_4_ ‘AND’ *M*_5_; *M*_2_ is caused by *M*_6_ ‘OR’ *M*_7_ ‘OR’ *M*_8_ ‘OR’ *M*_9_ ‘OR’ *M*_10_; and *M*_3_ is caused by *M*_11_ ‘AND’ *X*_43_.

$$\begin{array}{ll}{\rm{Accordingly}},\,\,{{M}}_{1} = {{M}}_{4}\, \times \, {{M}}_{5} \\ \qquad\qquad\qquad{\rm{M}}_{2} = {{M}}_{6}\, + \, {{M}}_{7}\,+\,{{M}}_{8}\, +\,{{M}}_{9}\, +\, {{M}}_{10}\\ \qquad\qquad\qquad{{M}}_{3} = {{M}}_{11} \, \times\,{{X}}_{43}\end{array}$$

We simplify the fault tree diagram of facial data breach by Boolean algebra as follows:

$${T} = {M}_{1} \, \times\, {M}_{2} \,\times\,{M}_{3} = ({M}_{4}\,\times\,{M}_{5})\, \times\, ({M}_{6}\,+\,{M}_{7}\,+\,{M}_{8}\,+\,{M}_{9}\,+\,{M}_{10})\,\times\, ({M}_{11}\,\times\,{M}_{43})$$

After step-by-step analysis, the bottom combination which cannot be decomposed constitutes the MCS, or the most basic path to the top event. Finally, we obtain 1224 MCSs, indicating that the current facial data system is extremely vulnerable and weak.

#### Analysis of structural importance

The structural importance analysis serves to assess the importance of each basic event from the fault tree diagram. The occurrence of each basic event has an impact on the top event, but the degree of impact is different. We conduct a structural importance analysis to clarify the degree of impact of each basic event, and we then rank each event according to impact degree. In this way, we can prioritise the events when taking safety precautions and ensure that the system remains economical, effective and safe. As a method of qualitative importance analysis, structural importance analysis is easy to perform and interpret, especially when quantitative data is absent.

There are two means to analysing structural importance: the first is to accurately calculate the structural importance of each basic event and then arrange it from large to small, but this is complex and cannot be carried out when the fault tree is large. The second is to approximately calculate the importance of each basic event according to the MCS. The following four principles should be followed when using the second approach (Barlow and Proschan, 1975):

1. The structural importance of the basic event in a single MCS is the largest.
2. All basic events that appear only in the same MCS have the same structural importance.
3. The degree of structural importance of each basic event that appears in several MCSs with the same number of basic events depends on the number of occurrences; that is, the number of occurrences is low and the structural importance degree is small. The number of occurrences is greater, its structural importance degree is also large. The occurrence times are equal, and the structural importance degree is equal.
4. If two basic events occur in MCSs with a different number of basic events, their structural importance degree is determined according to the following conditions: ① if their occurrence frequencies in the MCSs are equal, the structural importance degree of basic events in the MCSs with few events is greater. ② If the events appear less often in the MCS with few events and more often in the MCS with multiple events, they can be approximately calculated by the following formula (Zhang and Cui, 2002):

$$\mathop {\sum }\nolimits^ I\left( i \right) = \mathop {\sum }\limits_{Xi \in Kj} \frac{1}{{2^{n_i - 1}}}$$

where *I*(*i*) represents the approximate value of structural importance degree of basic event *Xi*; *Xi* ∈ *Kj* represents the basic event *Xi* belongs to the MCS of *Kj*; and *n*_*i*_ represents the number of basic events in the MCS where the basic event *Xi* is located.

Based on this principle, the structural importance analysis of the basic event of a facial data breach is carried out. Combined with the fault tree diagram, it is found that *X*_43_ is a single event that lies at the third level, which is the highest level among all basic events. Therefore, the importance of the basic event *X*_43_ is the largest. *X*_7_ appears twice in the fourth level of the fault tree diagram. The number of occurrences is higher, and its structural importance degree is accordingly large; therefore, *X*_7_ has the second greatest importance. According to principle (4), if the occurrence frequencies in the MCSs are equal, the structural importance of basic events in the MCSs with few events is greater. Compared with (*X*_44_, *X*_45_, *X*_46_, *X*_47_), (*X*_1_, *X*_2_,*X*_3_) and (*X*_4_, *X*_5_, *X*_6_) have fewer basic events, and the importance rank shall be *X*_1_ = *X*_2_ = *X*_3_ = *X*_4_ = *X*_5_ = *X*_6_ > *X*_44_ = *X*_45_ = *X*_46_ = *X*_47_. Likewise, *X*_44_ = *X*_45_ = *X*_46_ = *X*_47_ > *X*_40_ > *X*_8_ = *X*_9_ = *X*_10_ = *X*_11_ = *X*_12_ = *X*_13_ = *X*_14_ = *X*_15_ = *X*_16_ = *X*_17_ = *X*_18_ = *X*_19_ = *X*_20_ = *X*_21_ = *X*_22_ = *X*_23_ = *X*_24_ = *X*_25_ = *X*_26_ = *X*_27_ = *X*_28_ = *X*_29_ = *X*_30_ = *X*_31_ = *X*_32_ = *X*_33_ = *X*_34_ = *X*_35_ = *X*_36_ = *X*_37_ = *X*_38_ = *X*_39_ = *X*_41_ = *X*_42._

Finally, the rank of structural importance of each basic event can be obtained as follows:

*X*_43_ > *X*_7_ > *X*_1_ = *X*_2_ = *X*_3_ = *X*_4_ = *X*_5_ = *X*_6_ > *X*_44_ = *X*_45_ = *X*_46_ = *X*_47_ > *X*_40_ > *X*_8_ = *X*_9_ = *X*_10_ = *X*_11_ = *X*_12_ = *X*_13_ = *X*_14_ = *X*_15_ = *X*_16_ = *X*_17_ = *X*_18_ = *X*_19_ = *X*_20_ = *X*_21_ = *X*_22_ = *X*_23_ = *X*_24_ = *X*_25_ = *X*_26_ = *X*_27_ = *X*_28_ = *X*_29_ = *X*_30_ = *X*_31_ = *X*_32_ = *X*_33_ = *X*_34_ = *X*_35_ = *X*_36_ = *X*_37_ = *X*_38_ = *X*_39_ = *X*_41_ = *X*_42._

By comparing and analysing the occurrence frequency of the basic events in the MCS, it can be found that ‘Lack of laws and regulations’ (*X*_43_) and ‘Immaturity of face recognition technology’ (*X*_7_) have the greatest structural importance, which are important factors for a facial data breach. Facial data breach is a comprehensive issue involving multiple stakeholders, including technology companies, data platforms and various government departments. At present, it is difficult to clarify and agree on the lack of supervision and which jurisdiction the leakage of facial data belongs to, so it is future critical work to clarify the regulatory authority and regulatory responsibilities as well as to establish a unified and authoritative regulatory body and a multi-sector coordination mechanism. Particularly since the COVID-19 pandemic, face recognition technology has been widely applied, but the technology is far from mature, and there is a lack of corresponding regulatory rules and standards. Considering these vulnerabilities, many countries have issued national technology development strategies and formulated laws, regulations and national standards to monitor the development and application of facial recognition technology. In addition, the events related to individual consciousness (*X*_1_, *X*_2_, *X*_3_) and unsafe behaviour (*X*_4_, *X*_5_, *X*_6_) also have greater structural importance, demonstrating that the individual plays a vital role in personal data protection. Therefore, all related parties should jointly carry out public education on ‘Personal greed for small gains’ (*X*_1_), being ‘Incautious about the related products’ (*X*_2_) and the fact that ‘Individuals lack deletion consciousness’ (*X*_3_) to comprehensively improve individuals’ awareness of data protection and further avoid personal unsafe behaviours, such as ‘Individuals use simple password’ (*X*_4_), ‘Individuals upload facial data for entertainment actively and casually’ (*X*_5_) and ‘Individuals download unapproved apps’ (*X*_6_).

## Discussion

In this work, we identify potential and traditional breach risks of personal physiological data and assess the degree of importance of risk factors based on the fault tree diagram of facial data breach cases. We are also interested in proposing measures to reduce risks and opening up more avenues for building a robust data system for personal physiological data security.

First of all, the risk sources identified can effectively help identify various risks of physiological data breaches, providing some reference for relevant parties to conduct privacy and data protection. We take facial data breaches as the top event, collect related facial data breach cases, and identify the risks of cases combined with the risk causes of facial data breaches that are directly observed in the news reports and the expert analyses. Based on the risk identification, we list the 24 intermediate events and 47 basic events according to a logical relationship and thus draw a complete fault tree diagram of facial data breaches. According to the fault tree diagram of facial data breaches, we identified 1224 MCSs leading to the top event, demonstrating that facial data is vulnerable and can easily be breached. In the IoE era, personal physiological data is collected, stored and used on a large scale by various Internet service providers, which poses a significant risk of leakage. To some extent, this will seriously threaten the privacy and security of individuals (Li, 2020). Then, through the calculation of the structural importance based on the MCS, we obtained the importance rank of each basic event. The results indicated that ‘Lack of laws and regulations’ (*X*_43_), ‘Immature face recognition technology’ (*X*_7_), ‘Personal greed for small gains’ (*X*_1_), ‘Incautious about the related products’ (*X*_2_), ‘Individuals lack deletion consciousness’ (*X*_3_), ‘Individuals use the simple password’ (*X*_4_), ‘Individuals upload facial data for entertainment actively and casually’ (*X*_5_) and ‘Individuals download unapproved apps’ (*X*_6_) are the main factors leading to a facial data breach. In addition, in the context of IoE, we have also found some potential breach risk sources that have been ignored by previous studies, such as devices on physical attack surfaces and unsafe data transmission in cloud environments. Aiming at the emerging potential risk sources, the related parties need to update the risk knowledge and take targeted preventive measures to better protect privacy and data security.

Then, we have found that three types of events most easily lead to facial data breach: ‘Risk caused by individual’ (*M*_1_), ‘Risk during data management’ (*M*_2_) and ‘Supervision absence’ (*M*_3_), because they are the three intermediate events closest to the top event. Facial data breach is an integrative problem, and its complexity indicates that a breach is closely related to various factors, such as individuals, data management and legal supervision. Firstly, the disclosure of personal physiological data is closely related to personal active online behaviour. The prevalence of social media and the ubiquity of biometric technology have jointly subverted our way of life (Isaka and Adamu, 2022). Individuals need to use symbols that can identify themselves to promote and show themselves to society. In this process, personal privacy regarded as an important part of digital personal identity will be actively spread. Secondly, data management presents more intensive branches, indicating that data management involves the most breach problems. Primary data is not static and can be changed. Data undergoes a variety of actions for various purposes, including collection, storage, transmission, usage and destruction, which forms the data lifecycle and is difficult to manage. Each data lifecycle model has its own specific focus and risks (Alshammari and Simpson, 2017). Below the data management, the periods of data collection, data storage and data usage are the intermediate events that incur the most risk, indicating that there are many management vulnerabilities in the collection, storage and usage of physiological data. Moreover, a proactive regulator is a significant attribute in privacy security and data protection (Almeida et al., 2022). ‘Supervision absence’ (*M*_3_) can easily lead to problems such as shifting responsibility, the excessive power of the platform, and difficulties in safeguarding personal rights, which increases the occurrence of data breach events and the severity of risks (Almeida et al., 2022). In recent years, with the popularisation of biometric technology, the supporting regulatory policies and practical operation procedures have not been fully implemented. Although the EU has begun to implement GDPR known as the strictest data act, most countries are still in the exploratory stage. Taking face recognition technology as an example, there are great disputes between several states in the United States and European countries on whether to allow the use of this technology, which reflects the difficulty of legislation and regulation of this technology. Physiological data breach is a comprehensive issue involving multiple stakeholders, including third parties, technology companies, data platforms and various government departments. At present, it is difficult to clarify and agree on the lack of supervision and which jurisdiction the leakage of physiological data belongs to. Overall, the large-scale application of physiological data closely related to personal privacy is a worrying problem. During the data fluid procedure, there are more personnel and processes involved and invisible data breach risks are increased accordingly. Privacy has been placed in the dynamic practice of highly networked social structure, and individuals are increasingly difficult to control the flow and boundary of privacy.

We have assessed the structural importance of each basic event. The basic events are located at the end of each branch of the fault tree, and they are the initial causes of the top event. According to the structural importance of each basic event, it can effectively prevent the occurrence of breach events, timely discover hidden information such as the severity, macro situation, and driving factors of breach events (Ruijters and Stoelinga, 2015), and provide more reliable decision-making support for maintaining the safety of physiological data.

Firstly, we find that ‘Lack of laws and regulations’ (*X*_43_) has the greatest structural importance, which is an important factor for facial data breaches. As individual awareness of data rights is booming, especially due to the COVID-19 pandemic, the formulation of special legislation for personal information protection has become an international demand. Personal information protection rules have been issued all over the world, including the GDPR in the European Union, the California Privacy Rights Act in the US, and the Civil Code and Personal Information Protection Law in China. In 2022, personal information legislation and law enforcement were further promoted, and the proportion of the global population whose personal information is protected will increase from 10% in 2020 to 65% in 2023 (Gartner, 2020). However, the effectiveness of protecting physiological data is unknown due to the absence of specific and unified personal physiological data protection legislation, with some regional laws providing only very basic rights for accountability (Almeida, et al., 2022; Raposo, 2022). Therefore, it is suggested that national agendas should fast-track the process of special data supervision rules, especially focusing on addressing the problems including ‘Lack of collection standards and specifications’ (*X*_44_), ‘Lack of implementation rules for face recognition technology’ (*X*_45_) and ‘The review mechanism of science and technology ethics is incomplete’ (*X*_46_). In addition, special legislation related to personal physiological data protection should also be forward-looking to improve ‘The supporting policies not keeping up with technology’ (*M*_11_). The rapid development of information technology and its profound shaping of society have made data governance increasingly integrated with technology governance. OECD, OECD Digital Economy Outlook 2020 (2020) shows that countries generally recognise that adapting to information technology iteration is the current biggest challenge in data protection legislation and privacy regulations. The data protection laws that apply in the domain of emerging biometric technologies could provide a framework to alleviate individuals’ concerns about how their physiological data is used.

Similarly, how to effectively regulate and advance ‘Immature face recognition technology’ (*X*_7_) has also become a major issue that requires attention. Biometric recognition technology, especially face recognition technology has become widely used in various sectors. It can collect personal data remotely due to its non-contact characteristics, which leads to concerns regarding technical security, personal data disclosure and privacy violation. At present, many platforms publicly refuse to use biometric recognition technology; for example, in November 2021, Facebook claimed it would no longer use facial recognition technology to identify photos. However, the technology itself is not the main problem; if used and developed appropriately, this technology can bring great value to society. Due to the current rapid development of biometric technology, it is complex and highly uncertain, regulation in terms of legislation, government, industry and technology is advisable. In legislation, legal guarantees should be improved to clarify the application boundaries of biometric recognition by setting ‘franchise’ and entry standards that ensure the security of data information. The government should handle physiological data openly and transparently to ensure the right to access and modify personal physiological data. In terms of industry, the main responsibilities of the industry should be clarified, and the innovation of biometric recognition technology should be encouraged. Regarding the technology itself, the technological protections and technological norms should be strengthened and improved through, for example, encryption protection and authentication. All regulations will contribute to ensuring the security of personal data and promoting the maturity of biometric recognition technology.

Individual errors are the main cause of data breaches and individuals play a vital role in personal data protection (Boxcryptor, 2021). At present, individuals have insufficient awareness of personal privacy protection, they are accustomed to relying on Internet services, and lack an adequate understanding of privacy and awareness of risks. Despite individuals claim the importance of privacy protection, their actual behaviour often belies the importance. Scholars define this gulf between self-reported privacy attitudes and actual privacy behaviours as a privacy paradox (Hargittai and Marwick, 2016). Existing studies mostly explain the privacy paradox based on privacy computing and privacy fatigue (Choi et al., 2018). In the context of the IoE, the risk of personal physiological data breach is usually long-term and hidden, while the immediate interest temptation is direct, individuals usually give up their right to privacy control in order to obtain more personalised services. In addition, faced with complex terms, constantly changing settings and the prevalence of data breaches (Hargittai and Marwick, 2016), feelings of cynicism (Hoffmann et al., 2016), insensitivity (Moritz et al., 2021) or apathy (Hargittai and Marwick, 2016) could make individuals more passive and resigned, and choose not to read or agree directly (Choi et al., 2018). Individuals as the data subject should take the initiative to infiltrate the awareness of maintaining privacy and data security into daily life and reflect on their ‘interweaving with social practice’ (Seubert and Becker, 2019). Moreover, in the highly connected and networked IoE era, the awareness and ability of individuals to control their personal privacy are compromised by structural violations of privacy. Privacy protection is not an individual process, but rather a collective effect (Hargittai and Marwick, 2016), requiring governments, technology companies, individuals, and other privacy-related participants to continuously adjust policies, regulations, and behaviour patterns in the practice of economic development and technological innovation, in order to achieve privacy protection and comprehensive human development.

Finally, we also found the hidden risk sources of personal physiological data breaches in the IoE terminals and cloud. The IoE terminals are in the perception layer of the IoE, such as smartphones, wearable devices, sensors. Personal data is collected by perception layers in terminals, and then transmitted through the complex network, and finally stored in the server of the cloud service provider. In the context of IoE, the interaction between the IoE terminal devices and the cloud environment will lead to more personal data breach risk sources (Li, 2020; Fosch-Villaronga et al., 2018). First, devices with limited resources are difficult to deploy more advanced security mechanisms (such as complex encryption algorithms) (Iqbal et al., 2016), which can not only be connected by owners but also be intercepted by attackers (Mahmoud et al., 2015). In this case, an attacker can gain access to and control the device and may manipulate or extract data, control or interrupt the service. Second, many terminal devices generally exist in an open, untrusted and unmonitored physical environment, such as traffic control cameras, cloud robotics and environmental sensors exposed to the outside, maybe damaged artificially (Fosch-Villaronga et al., 2018; Iqbal et al., 2016). Third, cloud platforms open personal data to third parties, which also easily leads to personal data breaches. In the future, edge computing or fog computing architecture of ‘digital individuals’ will be an effective mechanism for data protection in the IoE era (Dang et al., 2019). In this architecture, the terminal device responsible for sensing physical objects sends personal data to the upper layer for processing and storage but does not need to upload all personal data to the cloud in real-time, which to a certain extent allows users to control their personal physiological data independently and reduce personal physiological data breach risks from the source.

## Conclusion

In the IoE environment, the emergence of a series of data technologies such as multimedia, biometrics, cloud computing and data mining has opened a new set of possibilities for innovative services and applications. However, this technology also introduces a complex scenario that must be efficiently managed to protect data security. In this scenario, the identification of personal physiological data risks is key to ensuring privacy and data security in the IoE environment. This study provides an important practical approach to revealing the occurrence and evolution mechanism of personal physiological data breaches and to implementing privacy protection measures by taking facial data breaches as a research object. Based on the findings, we maintain that personal physiological data breach has become a key security issue that deserves more attention in the theoretical and practical arenas. Overall, the core contributions and innovations of this research can be summarised into three aspects.

1. In terms of method, this study firstly attempts to use fault tree analysis to analyse facial data breaches, which is feasible and innovative in method. The security problem of physiological data systems is not caused by one aspect, and there are various relationships between many security risks. However, few studies have used systematic and objective methods to study and explain personal physiological data breach risks and their logical relationship. It is more comprehensive and reasonable to explore the logical relationship between risk sources at a deeper level in combination with real cases and to understand the importance of various factors in the whole risk system to then take well-directed measures.
2. In terms of practice, the construction of the fault tree diagram of a facial data breach can help the data management parties track the development of the data breach system and provide a knowledge base for risk identification. On the one hand, the knowledge base of risk sources can provide support for the identification of risk sources of subsequent physiological data breaches; On the other hand, the causal relationship is used to clarify the structural characteristics of the accident system, which is convenient for the control of the panoramic situation of physiological data breach and the oversight of the breach risk sensitive nodes. Moreover, it is of practical significance for improving the ability of risk adaptive governance and supporting the manageability and traceability of physiological data from data collection to data destruction.
3. In terms of theory, this study explores the plight of physiological data in the IoE era, providing a reference for privacy protection under the new situation. This study confirms that in a highly networked environment, a physiological data breach is a systematic process, so privacy protection is not a personal process, but a collective effort. In addition, it is found that the security of physical devices and the complexity of the cloud environment have triggered some new risk sources, providing ideas for building an effective mechanism for personal privacy protection in the future IoE era and enriching the relevant theories of privacy management.

## Data Availability

The data used in this article has been included in the article.

## Appendix 1 The details of 22 cases

**Table:**

NO.	Time	Case	Risk	Expert analysis	URL
1	2019	New data breach has exposed millions of fingerprint and facial recognition records	Simple user name and password; Access rights configuration error; Data exiting	–	https://www.forbes.com/sites/zakdoffman/2019/08/14/new-data-breach-has-exposed-millions-of-fingerprint-and-facial-recognition-records-report/?sh=2f1bbaf546c6
2	2020	Clearview AI data breach exposes facial recognition firm’s client list	The hacker; Individuals upload facial data for entertainment actively and casually, which are crawled;	Lack of collection standards and specifications	https://www.cpomagazine.com/cyber-security/clearview-ai-data-breach-exposes-facial-recognition-firms-client-list/
3	2019	In 2019, it was reported that hackers breached Apple’s iPhone Face ID user authentication in just 120 seconds	The hacker; Immature face recognition technology	–	https://www.analyticsinsight.net/what-will-happen-when-a-facial-recognition-firm-is-hacked/
4	2021	A group of hackers breached popular surveillance and facial recognition camera company, Verkada	Hacker attack; Illegal collection by offline face recognition camera	–	https://www.analyticsinsight.net/what-will-happen-when-a-facial-recognition-firm-is-hacked/
5	2020	Facial data of thousands of Chinese students has been leaked	Data outsourcing, hosted by a third party company	Lack of laws and regulations; Lack of implementation rules for face recognition technology； Insufficient safety education	https://baijiahao.baidu.com/s?id=1656340231011283716&wfr=spider&for=pc
6	2020	Residential neighbourhoods across China are adopting facial recognition	Incorrectly configured databases; Insufficient safety education	–	https://www.scmp.com/abacus/tech/article/3104512/facial-recognition-data-leaks-rampant-across-china-covid-19-pushes
7	2019	LLC, transferred copies of CBP’s biometric data, such as traveler images, to its own company network	Malware attack; Remote access by operators leaves data on other devices; The improper download and storage of database data	–	https://www.oig.dhs.gov/sites/default/files/assets/2020-09/OIG-20-71-Sep20.pdf
8	2021	Biometric Information Exposed in Slot Machine Operator Data Breach	Malware attack; Illegal storage of data by third parties; Illegal transfer of copies by third parties	–	https://findbiometrics.com/biometric-information-exposed-in-slot-machine-operator-data-breach/
9	2021	7–11 breached customer privacy by collecting facial imagery without consent	Low server security; App illegally opens cameras of mobile phone and other devices to collect data;	Lack of deletion consciousness; Lack of collection standards and specifications	https://www.zdnet.com/article/7-eleven-collected-customer-facial-imagery-during-in-store-surveys-without-consent/
10	2021	Consumer-facing Companies Still Have Few Incentives to Stop Data Breaches, and That’s a National Security Concern	Operators steal data	The enterprise has ineffective security measures and insufficient data protection capacity	https://www.cfr.org/blog/consumer-facing-companies-still-have-few-incentives-stop-data-breaches-and-thats-national
11	2019	Massive biometric data breach found in system used by banks and Met police	The database without password; Web crawler collection	The physically collection device is invaded physically	https://www.itpro.co.uk/data-breaches/34206/massive-biometric-data-breach-found-in-system-used-by-banks-and-met-police
12	2021	Students sue online exam proctoring service ProctorU for biometrics violations following data breach	The third-party did not delete the data as required; The review mechanism of science and technology ethics is incomplete; Lack of data deletion mechanism	–	https://lawstreetmedia.com/news/tech/students-sue-online-exam-proctoring-service-proctoru-for-biometrics-violations-following-data-breach/
13	2021	Hackers Breach Thousands of Security Cameras, Exposing Tesla, Jails, Hospitals	An international hackerUnauthorized access	–	https://www.bloomberg.com/news/articles/2021-03-09/hackers-expose-tesla-jails-in-breach-of-150-000-security-cams
14	2019	The SenseNets Horizon company leaks billions of facial data	The database without password；Individual’s insufficient security awareness; Database security protocol flaw	Illegal trade; The enterprise has ineffective security measures and insufficient data protection capacity; The database without password; Access rights configuration error	https://baijiahao.baidu.com/s?id=1625635651342970434&wfr=spider&for=pc
15	2019	IBM collects online photos without consent, the individual find it impossible to delete their facial data	The management failed to delete the data as required Hacker attack	–	https://www.nbcnews.com/tech/internet/facial-recognition-s-dirty-little-secret-millions-online-photos-scraped-n981921
16	2021	Everalbum collects facial pictures illegally	App illegally opens cameras of mobile phone and other devices to collect data; The management failed to delete the data as required	Lack of laws and regulations; Lack of implementation rules for face recognition technology	https://www.latimes.com/business/story/2021-01-29/column-facial-recognition-privacy
17	2020	Facial data leakage of students in some schools in Sichuan and Gansu	Unsafe database; Illegal storage of data by third parties	–	https://baijiahao.baidu.com/s?id=1656340231011283716&wfr=spider&for=pc
18	2020	Zhang Fu and others violated citizens’ personal information and fraud	Commercial companies actively sell data; Immature face recognition technology	–	https://www.sohu.com/a/373482018_120032
19	2021	Abuse of face recognition in stores such as Kohler bathroom and BMW	Concealed collection means; Rough collection method; Illegal trade	Lack of laws and regulations; Lack of implementation rules for face recognition technology	https://www.sohu.com/a/456089454_393779
20	2019	Twinning’s personal information breach	Incautious about the related products; Incorrectly configured databases; The physically invaded collection device	–	http://www.woshipm.com/ai/1844718.html
21	2019	The biometric data leakage occurred to Suprema	Not desensitized core data; Data deletion incompletely; The physically compromised storage device	Commercial companies went bankrupt and data were resold	https://us.norton.com/internetsecurity-emerging-threats-biometric-data-breach-database-exposes-fingerprints-and-facial-recognition-data.html
22	2020	Abuse of face recognition technology in housing sales industry (29 Administrative Punishment Cases)	Concealed collection means; Illegal transfer of copies by third parties	Lack of implementation rules for face recognition technology; The review mechanism of science and technology ethics is incomplete	https://m.thepaper.cn/baijiahao_10148531
